# Supplementary material for: COZOID: contact zone identifier for visual analysis of protein-protein interactions
Source: BMC Bioinformatics. 2018 Apr 6;19:125. doi: 10.1186/s12859-018-2113-6 (PMC5889581; doi:10.1186/s12859-018-2113-6)
Supplement: Supplementary file 2 — Software build. Executable binary file of the software tool. (ZIP 118,784 kb) [file 12859_2018_2113_MOESM2_ESM.zip › cozoid/java/docs/beansbinding-1.2.1-doc.zip!/org/jdesktop/el/class-use/ValueExpression.html]

Uses of Class org.jdesktop.el.ValueExpression (Beans Binding)


---


|  |  |  |
| --- | --- | --- |
| **Overview** \| **Package** \| **Class** \| **Use** \| **Tree** \| **Deprecated** \| **Index** \| **Help** | |  |
| PREV   NEXT | **FRAMES**    **NO FRAMES**     **All Classes** |  |


---


## **Uses of Class org.jdesktop.el.ValueExpression**

| Packages that use ValueExpression | |
| --- | --- |
| **org.jdesktop.el** | Customized version of EL for Beans Binding; not for general use. |
| **org.jdesktop.el.impl** | Customized version of EL for Beans Binding; not for general use. |
| **org.jdesktop.el.impl.lang** | Customized version of EL for Beans Binding; not for general use. |

Uses of ValueExpression in org.jdesktop.el

| Methods in org.jdesktop.el that return ValueExpression | |
| --- | --- |
| `abstract  ValueExpression` | `ExpressionFactory.createValueExpression(ELContext context, java.lang.String expression, java.lang.Class<?> expectedType)`             Parses an expression into a `ValueExpression` for later evaluation. |
| `abstract  ValueExpression` | `ExpressionFactory.createValueExpression(java.lang.Object instance, java.lang.Class<?> expectedType)`             Creates a ValueExpression that wraps an object instance. |
| `abstract  ValueExpression` | `VariableMapper.resolveVariable(java.lang.String variable)` |
| `abstract  ValueExpression` | `VariableMapper.setVariable(java.lang.String variable, ValueExpression expression)`             Assign a ValueExpression to an EL variable, replacing any previously assignment to the same variable. |

| Methods in org.jdesktop.el with parameters of type ValueExpression | |
| --- | --- |
| `abstract  ValueExpression` | `VariableMapper.setVariable(java.lang.String variable, ValueExpression expression)`             Assign a ValueExpression to an EL variable, replacing any previously assignment to the same variable. |

Uses of ValueExpression in org.jdesktop.el.impl

| Subclasses of ValueExpression in org.jdesktop.el.impl | |
| --- | --- |
| `class` | `ValueExpressionImpl`             An `Expression` that can get or set a value. |
| `class` | `ValueExpressionLiteral` |

| Methods in org.jdesktop.el.impl that return ValueExpression | |
| --- | --- |
| `ValueExpression` | `ExpressionFactoryImpl.createValueExpression(ELContext context, java.lang.String expression, java.lang.Class expectedType)` |
| `ValueExpression` | `ExpressionFactoryImpl.createValueExpression(java.lang.Object instance, java.lang.Class expectedType)` |

Uses of ValueExpression in org.jdesktop.el.impl.lang

| Methods in org.jdesktop.el.impl.lang that return ValueExpression | |
| --- | --- |
| `ValueExpression` | `ExpressionBuilder.createValueExpression(java.lang.Class expectedType)` |
| `ValueExpression` | `VariableMapperImpl.resolveVariable(java.lang.String variable)` |
| `ValueExpression` | `VariableMapperFactory.resolveVariable(java.lang.String variable)` |
| `ValueExpression` | `VariableMapperImpl.setVariable(java.lang.String variable, ValueExpression expression)` |
| `ValueExpression` | `VariableMapperFactory.setVariable(java.lang.String variable, ValueExpression expression)` |

| Methods in org.jdesktop.el.impl.lang with parameters of type ValueExpression | |
| --- | --- |
| `ValueExpression` | `VariableMapperImpl.setVariable(java.lang.String variable, ValueExpression expression)` |
| `ValueExpression` | `VariableMapperFactory.setVariable(java.lang.String variable, ValueExpression expression)` |

---


|  |  |  |
| --- | --- | --- |
| **Overview** \| **Package** \| **Class** \| **Use** \| **Tree** \| **Deprecated** \| **Index** \| **Help** | |  |
| PREV   NEXT | **FRAMES**    **NO FRAMES**     **All Classes** |  |


---
